# Editorial: Women in cytokines and soluble mediators in immunity

**DOI:** 10.3389/fimmu.2024.1395165

**Published:** 2024-03-14

**Authors:** Diana Boraschi, Giselle Penton-Rol, Olukemi Amodu, Marita Troye Blomberg

**Affiliations:** ^1^ Laboratory Inflammation and Vaccines, Shenzhen Institute of Advanced Technology (SIAT), Chinese Academy of Sciences (CAS), and China-Italy Joint Laboratory of Pharmacobiotechnology for Medical Immunomodulation, Shenzhen, China; ^2^ Institute of Biomolecular Chemistry, National Research Council (CNR), Pozzuoli, Italy; ^3^ Stazione Zoologica Anton Dohrn, Napoli, Italy; ^4^ Center for Genetic Engineering and Biotechnology (CIGB), Playa, Cuba; ^5^ Department of Physiological Sciences, Professor of Immunology at the Latin American School of Medicine (ELAM), Havana, Cuba; ^6^ Genetics and Molecular Sciences Unit, Institute of Child Health, College of Medicine, University of Ibadan, Ibadan, Nigeria; ^7^ Department Molecular Biosciences, the Wenner-Gren Institute, Stockholm University, Stockholm, Sweden

**Keywords:** women, cytokines, soluble mediators, immunity, pathology, diagnosis, therapy

The Research Topic dedicated to “*Women in Cytokines and Soluble Factors in Immunity*” was launched in March 2022 by two experienced and two younger female scientists, active in four different continents. In 24 months, we have collected over 60 contributions from female scientists around the World. Most of the papers are original research studies and deal with human diseases (tumors, infections), evaluating the pathological or predictive role of cytokines, but also the importance of cytokines in invertebrate immune defense. Most of the papers list young PhD students as first author, but many very experienced female scientists have also contributed. Most of the papers come from Europe, but significant contributions were received from Asia, Americas and Africa. Impressively, almost one third of the papers are cross-continental collaborations. While much still must be done for promoting female participation to scientific research, in particular in developing countries, this Research Topic underlines an encouraging scenario of commitment and dedication to science, desire of collaboration, capacity to coach, train and support younger female scientists to engage in a scientific career.

## 1 Cytokines and soluble factors in immunity

The field of cytokines and other soluble factors in immunity encompasses many different scientific areas, from evolution of immunity to clinical diagnostic studies. This Research Topic includes several important contributions in different areas of basic and clinical research on soluble immune factors, as briefly summarized below.

### 1.1 Invertebrate immunity

Soluble immune factors are essential elements in the defensive system of invertebrate metazoans, and include enzymes such as lysozyme and phenoloxidase, complement and inflammatory cytokines such as IL-17 and TNF (1–6). A comprehensive analysis of soluble factors-mediated immunity in two different invertebrate phyla, annelids and bivalve mollusks, is reported by an excellent group of four women scientists active in two different European countries (Canesi et al.). Two studies from Italy and Italy-China analyzed the immune response of tunicates to biotic or abiotic challenges and showed that the modulation of several immune factors, including complement, cytokines, enzymes and variable chitin-binding and LPS-binding proteins, is the basis of primary and memory responses in *Ciona robusta* (Marino et al., Liberti et al.).

### 1.2 Mammalian immunity

In mammalian immunity, soluble factors have a major importance both for innate and adaptive immune response. As for invertebrates, complement is one of the key anti-bacterial effector systems (4, 7, 8). For adaptive immunity, antibodies are the highly specific and effective neutralizing tools (8). Cytokines and chemokines are soluble factors that participate to both innate and adaptive immunity, representing the natural bridge between the two systems (8). The role of soluble factors in the T cell development has been extensively examined, including at the level of intrathymic differentiation (8, 9). A very interesting study addresses the role of two such factors, IL-2 and IL-15, in driving the differentiation of distinct Treg cells in the thymus (Apert et al.). It is notable that such study is the result of a collaboration between three different European countries, France, Switzerland and UK. A comprehensive assessment of the multifaceted role of chemokines in immunity is provided by a young researcher active in one of the groups that discovered chemokines, in Switzerland (Cecchinato et al.). The ability of innate cells such as macrophages to produce cytokines and other soluble factors in response to challenges is generally assessed *in vitro* in culture systems that may not reproduce accurately the *in vivo* conditions (10, 11). This is a shortcoming that may lead to misleading results and inaccurate prediction of immune reactivities. A joint collaboration between Dutch, UK and German researchers pointed out how variations in cell density in culture can influence the function and cytokine production by human macrophages, which warrants a particular attention in the reliable design of our cell culture systems (Ruder et al.).

### 1.3 Cytokines/soluble factors’ profile in diseases as diagnostic/prognostic markers

The role of cytokines and other immune soluble factors in diseases has gained increasing importance, and many studies have pointed at the possibility of using them as diagnostic markers, able to identify the type and stage of multiple different diseases, and as prognostic tools for predicting future susceptibility or resistance to disease development.

#### 1.3.1 Infections

The recent COVID-19 pandemic has produced numerous studies of cytokines as markers of disease and disease severity. Association with mortality, as well as other immune and vascular biomarkers, is reported in two studies, one in Spain (Sanchez-de Prada et al.) and one in South Africa in collaboration with UK (Shaw et al.). The soluble immune factors’ profile in COVID-19 convalescent pregnant women was examined in an extensive Brazilian study encompassing ten different institutions (Fernandes et al.). The lung inflammatory conditions and the infection-dependent epithelial to mesenchymal transition was examined at the single cell level in a collaborative study between China and USA (Zhang et al.). The importance of the viral nucleoprotein in inducing anti-inflammatory innate memory was examined in a joint study between the European Commission JRC, Italy and China (Urban et al.). A very interesting study in Cameroon in collaboration with Italy highlighted the presence of antibodies reactive to SARS-CoV-2 in archive samples of HIV patients dating back to before the COVID-19 pandemic (Aissatou et al.). Inflammation-related soluble factors were examined in HIV patients both for optimizing treatment in Cameroonian adolescents in a collaborative study between Cameroon, Italy, France and South Africa (Ka’e et al.) and for predicting hematological complications and, again, optimize treatment, in a Danish study (Grønbæk et al.). Response to viruses has been further examined in a collaborative Brazil-Germany study in terms of systems biology dynamical evaluation of interferon-related signatures in Dengue infection (Usuda et al.) and single cell-based identification of the chemokine CCL5 as biomarker of macrophage response to DNA sensing in a USA work (McCarty et al.).

Several studies focused on parasitic diseases, in particular malaria. A comprehensive immunogenomic study was performed in a collaboration between USA, Mali and Portugal to identify an immune profile predictive of susceptibility to clinical malaria (Mbambo et al.). Another study from Nigeria explored the possible pathogenic role of TNF-α in malaria patients with type 2 diabetes (Ademola et al.). In a study from the Côte d’Ivoire, another inflammatory cytokine, IL-1β, and other members of the IL-1 family, were examined in sickle cell disease in Sub-Saharan Africa, in the attempt to design a better management of the disease (Siransy et al.). The immunological aspects of clinical forms of cutaneous leishmaniasis in North Africa and French Guiana were addressed in a collaborative study between France, Tunisia and French Guiana (Saidi et al.).

Focus on bacterial infections encompasses a study examining the profile of cytokines and chemokines in people recently infected with *Mycobacterium tuberculosis*, within a collaboration between Colombia and Canada (Herrera et al.), a Canadian-Swedish study reported on correlates of protection in *Francisella tularensis* infection (Lindgren et al.), a very interesting evaluation of the osteoblast secretome in osteomyelitis induced by *Streptococcus aureus* was contributed by an Italian group (Granata et al.), and an Italian-UK collaboration provided an excellent review of the value of PTX3 as prognostic marker of mortality in sepsis (Davoudian et al.).

#### 1.3.2 Pregnancy

The involvement of soluble immune factors in some pathological aspects associated with pregnancy has been the focus of a number of manuscripts. The levels of cytokines in patients undergoing procedures for *in vitro* fertilization were examined in a study from Poland (Piekarska et al.). The cytokine levels in first trimester-pregnant women developing hypertension after assisted reproductive technology were examined in a Chinese study (Lan et al.), while another Chinese study addressed the association between circulating inflammatory cytokines and pregnancy-associated hypertension (Guan et al.). Pregnancy-associated immunomodulation through extracellular vesicles and soluble factors released by amniotic mesenchymal stromal cells was investigated for possible translational applications by an Italian study (Papait et al.). The correlation between immune parameters, including cytokines, and cervical insufficiency and preterm births was addressed by a Korean study (Jeong et al.). Eventually, the association between monocyte signatures and the risk of developing chronic lung disease in preterm infants was shown in a German study (Windhorst et al.).

#### 1.3.3 Other conditions

Cytokines and soluble factors can act as markers of disease, disease progression and disease severity in several diseases in which inflammation is involved. A collaborative study between Germany and Austria addressed the presence of systemic inflammation-related markers in polyneuropathies (Garcia-Fernandez et al.), while a study from Qatar has investigated the cellular sources of anomalous cytokine levels in autism (Nour-Eldine et al.). In a Finnish/USA study, circulating IL-21 was found to correlated with disease progression in type 1 diabetes (Schroderus et al.), whereas a Polish study suggested adipokines could be disease biomarkers in type 2 diabetes, demonstrated in a cat model (Sierawska and Niedźwiedzka-Rystwej et al.). Soluble prognostic and diagnostic biomarkers of osteoporosis were investigated in a Chinese study (Wang et al.). In an Italian study, soluble biomarkers of disease mechanisms and phenotypes of systemic sclerosis were identified with an aptamer-based proteomic approach (Motta et al.). The distribution of IL-22BP in Crohn’s disease was the focus of a French investigation (Fantou et al.). Eventually, the contribution of mast cell proteases and IL-33 processing in the development of allergic lung inflammation was examined in a mouse model in a collaborative study between Belgium, Austria, Russia, Germany and China (Krysko et al.).

### 1.4 Cytokines and soluble factors in pathogenic mechanisms

Whether anomalies in cytokine production is a consequence of disease or may be part of the pathogenesis is still an open question. Several studies included in this collection tackle the issue in the attempt to identify the involvement of cytokines in pathogenesis vs. pathology. Several of the studies focused on cytokines of the IL-1 family, which include highly inflammatory factors, such as IL-1β, that have a well-known role in many chronic inflammatory and degenerative diseases (12, 13). A manuscript from Italy and China examined the role of IL-1 family cytokines in neuroinflammatory and neurodegenerative diseases and concluded that most likely these cytokines are involved in both pathogenesis and downstream pathological symptoms (Boraschi et al.). That IL-1β, the best-known inflammatory cytokine of the family, can be involved in tumorigenesis is known (14, 15), but a study from Singapore and Switzerland further identified AIM2 activation as one of the factors contributing to the production of IL-1β in the tumor microenvironment (Chew et al.). IL-1α, a cytokine mostly present as membrane bound molecule thereby acting through cell-to-cell contact (16, 17), was found responsible for activating leukocyte adhesion during atherogenesis in a German study (Maeder et al.). Another German study found that IL-36γ, a less known inflammatory cytokine of the IL-1 family mostly active at the tissue level, is responsible for interferon-independent liver injury caused by the Rift Valley virus (Anzaghe et al.). Other specific cases were examined: the role of IL-11 in fibrosis in interstitial lung disease, studied using human organoids by a German group (Kastlmeier et al.); the dysregulation of LIF in endometriosis, proposed by a Canadian-USA collaboration (Zutautas et al.); the role of GAS6/TAM in regulating NK cell recognition of multiple myeloma cells and degranulation studied by an Italian-USA group (Kosta et al.); and the involvement of the complement terminal complex in the inflammatory activation of retinal pigment epithelium and the associated development of age-related macular degeneration proposed by a German collaboration study (Busch et al.). Eventually, a group of studies examined the entire spectrum of cytokines and soluble factors associated with initiation *vs*. maintenance of different pathologies. A collaborative study between Switzerland and Spain reviewed the contribution of cytokines to the hyperinflammatory spectrum (Planas et al.). A USA group reviewed the contribution of oncostatin M, a cytokine of the IL-6 family, in inflammatory diseases (Wolf et al.). In cancer, the contribution of tumor-associated macrophages and their soluble factors in lung metastatic melanoma was addressed in a China-Germany collaborative study (Xiong et al.). Eventually, an informative review on the role of cytokines in Acute Myeloid Leukemia was contributed by an Austrian group (Luciano et al.).

### 1.5 Cytokines and cytokine modulation in therapy

Targeting cytokines for curing diseases is an approach that has turned out to be successful in several instances, see as an example the TNF-α or IL-1β inhibitory biologicals currently used in several chronic inflammatory diseases (18–21), although the efficacy is not complete and side effects can be observed [such as the tuberculosis activation in patients with latent tuberculosis treated with anti-TNF-α drugs; (22)]. An historical excursus describing how several of these cytokine inhibitors were originally identified is provided by Daniela Novick, a pioneer woman scientist who accelerated substantially the development of anti-cytokine drugs (Novick et al.). Inflammation, including induction of inflammatory cytokines, has been for decades the core of cancer immunotherapy, with the use of bacteria and bacterial-derived molecules (23). The use of BCG is currently the golden standard treatment for non-invasive bladder cancer, and its effect includes the activation of an anti-tumor innate and inflammatory response (24, 25). Based on the notion that innate immunity is non-specific, a large collaborative study (involving researchers from Austria, The Netherlands, France, Italy, Russia, Poland and Hong Kong) showed that treatment of bladder cancer patients with BCG induced a systemic innate response able to counteract the SARS-CoV-2 infection (Pichler et al.). On the same line, a very interesting study from an Iranian and Canadian collaboration shows that the use of molecules that induce innate/inflammatory activation can substantially increase the therapeutic immune response against colon cancer in a preclinical model and also downregulates the pathological presence of fibroblasts in the tumor microenvironment (Haijabadi et al.). A review of the innate immunity in the airways, contributed by an Icelandic/Swedish group, comes to similar conclusions, *i.e.*, that activation of innate immune/inflammatory epigenetic mechanisms by bacterial products may contribute to effective response and disease resolution (Myszor and Gudmundsson).

Anti-inflammatory strategies are the most common approaches to cytokine inhibition in different diseases. In liver transplantation from brain dead donors, inflammation is largely dependent on increased IL-1β production and was shown, in a study from a Mexico-Spain-Uruguay collaboration, to be the main cause of rejection, as it could be mitigated by treatment with the IL-1 antagonist IL-1Ra (Casillas-Ramirez et al.). Interestingly, a German-UK study on IL-37, an anti-inflammatory cytokine of the IL-1 family that is active in the gut, showed that the IL-37 role at the level of the mucosal epithelium is minimal, suggesting an anatomically distinct and localized anti-inflammatory activity (Krohn et al.). Among anti-inflammatory strategies, the use of phycocyanobilin, a tetrapyrrole chromophore from microalgae, displays substantial anti-inflammatory properties and was found able to inhibit leukocyte infiltration and cytokine production in a rheumatoid arthritis model (Marin-Prida et al.) and in a multiple sclerosis model (Marin-Prida et al.), as reported by two Cuban studies, one in collaboration with Brazil, China, Mexico, and the other one with Brazil and Germany. A French study explored the use of a synthetic histamine analogue that binds to CXCR4 (a chemokine receptor important for leukocyte mobilization) for inhibiting inflammation in juvenile arthritis cells *in vitro* and in a mouse model *in vivo*. Using this model, they observed substantial reduction of inflammatory cytokines, immune cell infiltration and joint and bone erosion (Bekaddour et al.). Anti-inflammatory effects were also observed in a Cuban study using a modified peptide derived from HSP60 both in animal models of RA and in human patients The treatment inhibited inflammatory cytokine production, including IL-17, and ameliorated the cytokine storm in COVID-19 patients (Dominguez-Horta et al.). Anti-inflammatory strategies targeting T cells included the above-mentioned study, which identified an increase in Treg cells, and a collaborative study between Cuba, Korea and Portugal on a mutant of IL-2, which showed that the antitumor activity of the mutein is based on the change of the balance between CD8^+^ and Treg cells (Carmenate et al.). Eventually, an Italian study showed that a synthetic sulfolipid can decrease IL-12 production by dendritic cells and promote T cell differentiation towards Treg (Barra et al.).

## 2 Women in cytokines and soluble factors in immunity

**Figure 1:**
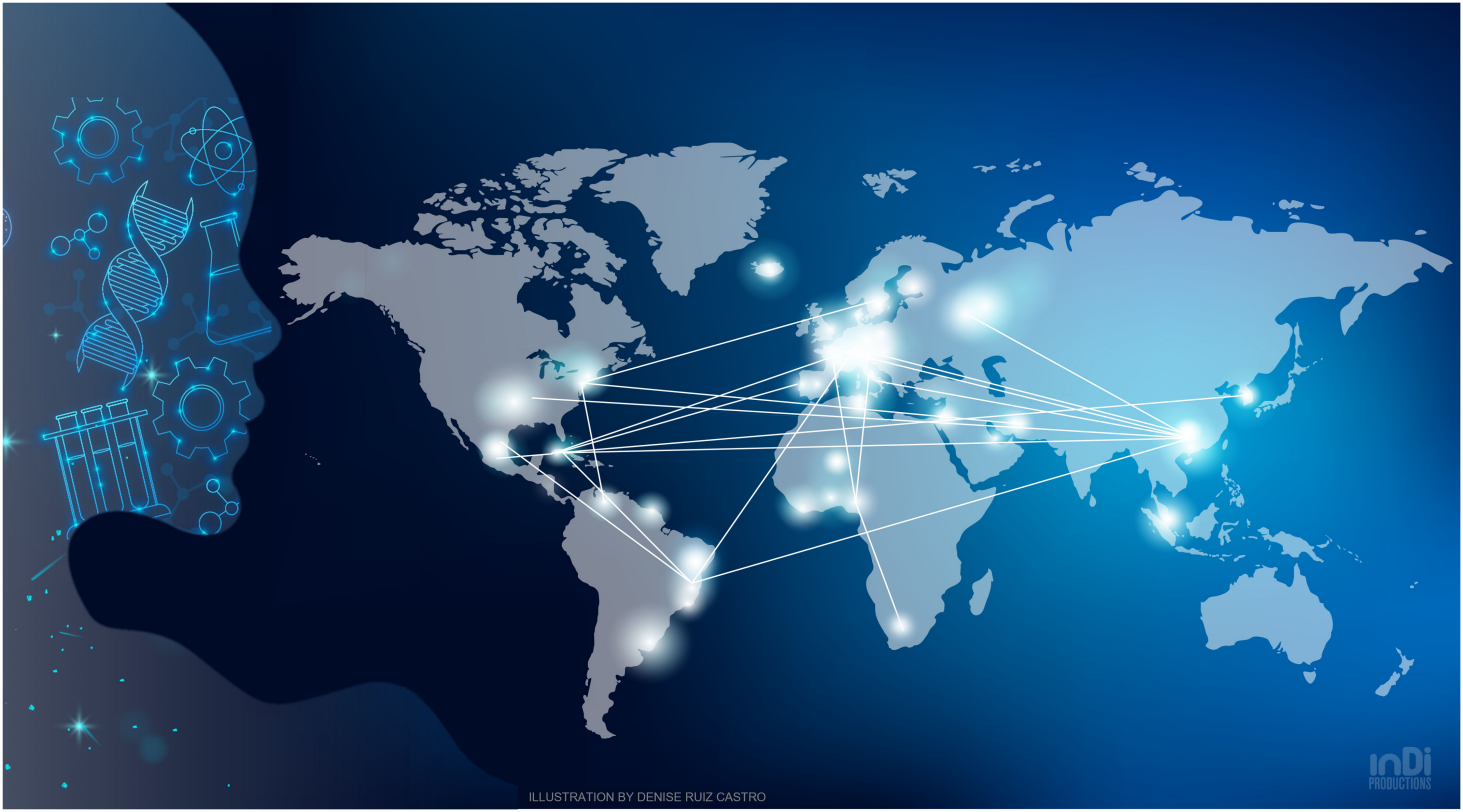
Worldwide distribution of the contributions to “Women in Cytokines and Soluble Factors in Immunity” with their collaborative interconnections. Artwork by Denise Ruiz Castro and Maykel Pedro Penton Martinez (Design project InDi productions, Havana, Cuba) and Sheila Delgado-Lora.

This Research Topic provides much more than a number of good scientific papers on the general topic of cytokines and soluble factors in immunity. Being dedicated to women immunologists active in the area of cytokines, the Research Topic aimed to be as inclusive as possible, having expert scientists who could set an inspiring example for the young students who are just approaching science, and encouraging female researchers to engage in a scientific career in every country, in particular those where research conditions are more difficult in general and for women in particular. To reflect our intention, the four editors of the Research Topic are two old and two young female immunologists, active in four different continents. We have collected 62 contributions, in which the first or the senior author are female scientists. Many are young PhD students, while others are very experienced researchers. Authors of these papers come from 40 countries across four continents (Europe, Asia and Middle East, Africa, Americas). Impressively, over 77% of the studies are collaborative studies, and 44% of these collaborations are intercontinental, as we did underline in the previous paragraphs. This reflects a very encouraging trend, *i.e.*, that scientists (and women in particular) strongly believe that progress means collaboration without borders and without any type of bias (**Figure 1**).

We had in 2023 two important examples of successful women, whom are worth mentioning for their life-long commitment. Claudia Goldin, an American economist, has been awarded the Nobel Prize in Economic Sciences for her relentless engagement in understanding and making known the gap between women and man in the labor market (26). Her work and her analysis of the reasons why the majority of undergraduates in USA are women are impacting substantially on the general understanding of the female working and social conditions, well beyond her country. Another inspiring example is Katalin Karicó, a Hungarian biochemist who moved to USA when she was 30, after she lost her lab position due to lack of funding. Katalin is an expert in RNA, and in 1989, soon after having moved to the USA, she started working on mRNA and focused on mRNA-based therapeutic approaches. In the following years, she encountered huge difficulties in obtaining funding and in developing her academic career but she never abandoned her focus on therapeutic mRNA, and eventually she succeeded in dumping the RNA inflammatory activity and devising an effective delivery system. This was the basis for the first widespread human application of mRNA, as vaccine for SARS-CoV-2. She was awarded the Nobel Prize in Physiology and Medicine in October 2023. Few months earlier, her biography was published as an illustrated children book, with the compelling title “Never give up” (27).

The contribution of women to science is substantial, but the gaps and inequalities are still outstanding in many countries and institutions. Although there are many initiatives aiming at raising awareness and encourage women’s participation in science (for instance the International Day of Women and Girls in Science, celebrated every February 11), we, as women scientists, are those who should “never give up”, both in pursuing advancement of knowledge and in closing gaps and eliminating inequalities.

## Author contributions

DB: Writing – review & editing, Writing – original draft. GP-R: Writing – review & editing. OA: Writing – review & editing. MB: Writing – review & editing.
